# Muscle health in the modern era of incretin‐based therapies

**DOI:** 10.1111/eci.70155

**Published:** 2025-12-02

**Authors:** Giuseppe De Girolamo, Moris Sangineto, Giuseppe Di Gioia, Rossella Bianco, Martina Ciarnelli, Gaetano Serviddio

**Affiliations:** ^1^ Internal Medicine and Liver Unit, Department of Medical and Surgical Sciences University of Foggia Foggia Italy; ^2^ Center for Human Nutrition Washington University School of Medicine St. Louis Missouri USA

**Keywords:** GIP, GLP‐1, GLP‐1RA, muscle loss, weight loss

## Abstract

**Background:**

Intentional weight loss improves obesity‐related outcomes but reduces lean body mass, raising concern about skeletal muscle mass, function and long‐term health. GLP‐1 receptor agonists (GLP‐1RAs) produce clinically meaningful weight loss primarily by lowering energy intake and slowing gastric emptying, with additional benefits on insulin sensitivity and inflammation.

**Objective:**

To clarify GLP‐1RAs' effects on skeletal muscle tissue, body composition and physical function by reviewing preclinical and clinical evidence.

**Findings:**

Across randomized and controlled studies, GLP‐1RAs reduce fat mass more than lean body mass. Functional measures appear preserved. Preclinical and early clinical data suggest improvements in muscle quality (microvascular recruitment, mitochondrial efficiency, reduced intramuscular fat) rather than hypertrophy.

**Conclusions:**

GLP‐1RA‐induced weight loss is largely fat mass with modest absolute lean body mass decline and no consistent deterioration in strength or function. Progressive resistance training, adequate/high‐quality protein and periodic monitoring of body composition and performance should accompany therapy, especially in higher‐risk patients.

## Graphical abstract

GLP‐1 receptor agonists produce weight loss predominantly from fat mass loss, with smaller reductions in lean mass, possibly impairing muscle performance. However, newer evidence suggests muscle function and quality are preserved, potentially via improved mitochondrial efficiency, increased microvascularization and lower intramuscular fat. Practical mitigation includes progressive resistance training, adequate protein intake and routine monitoring of body composition and functional outcomes.

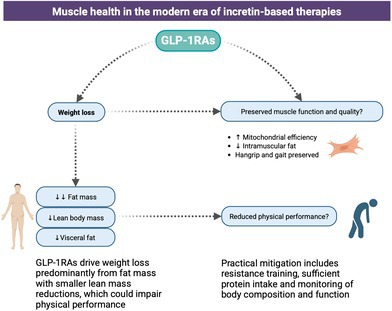

## 1 INTRODUCTION

Obesity is linked with a broad range of cardiometabolic diseases, including type 2 diabetes (T2D), atherosclerosis, dyslipidemia, metabolic‐associated fatty liver disease (MASLD).^1^ Modest (5%–10%) weight loss (WL) has been shown to successfully improve clinical parameters associated with obesity‐related diseases.^2^ However, WL typically reduces lean body mass (LBM) by ~25%,^3^ raising concerns about skeletal muscle mass (SMM), physical function and long‐term health. In fact, it is known that low muscle mass in obesity negatively impacts quality of life and correlates with an increased all‐cause mortality rate.^4^

Probably, the greater absolute amounts of LBM and SMM, due to higher mechanical loading,^5^ protect people with obesity from excessive decrease of SMM during intentional WL of about 10%, improving physical function.^6^ In other words, the relative decrease in SMM is less than the relative decrease in fat mass (FM), hence increasing the SMM to FM or weight ratio. WL also entails better muscle quality by decreasing intramuscular triglycerides, improving insulin sensitivity and glucose homeostasis and dampening muscle mass wasting.^7^, ^8^

**FIGURE 1:**
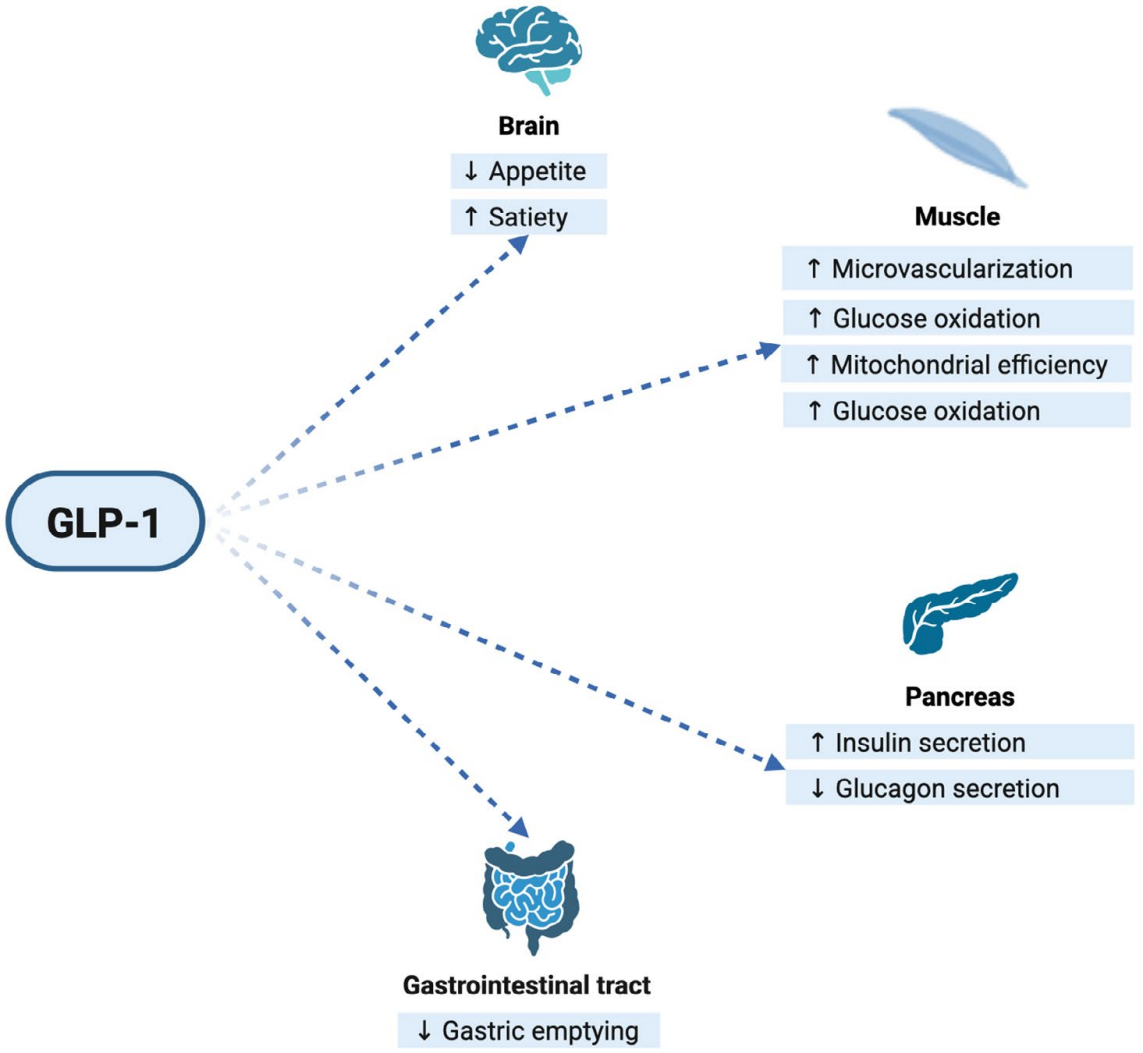
GLP‐1 acts on the brain to reduce appetite and increase satiety; on the pancreas to enhance glucose‐dependent insulin secretion and suppress glucagon; on the gastrointestinal tract to slow gastric emptying; and on skeletal muscle to improve microvascular recruitment, mitochondrial efficiency and glucose oxidation. Collectively, these pathways support glycemic control and may help preserve muscle quality; **↑**, increase; **↓**, decrease.

Glucagon‐like peptide‐1 receptor agonists (GLP‐1 RAs) have revolutionized obesity care, thanks to their ability to achieve clinically meaningful WL (15%–20%) after 1–1.5 years of therapy,^9^ emerging as a cornerstone therapy for the management of obesity and T2D. GLP‐1RAs promote WL primarily by reducing energy intake through central effects and slowing gastric emptying, while meal‐dependent insulin secretion increases (Figure 1). Taken together, these effects drive a sustained negative energy balance, capable of inducing WL.^10^ Acting in hindbrain and hypothalamic circuits to reduce appetite and food reward, GLP‐1RAs and dual incretin therapy can reduce ad libitum energy intake typically by ~16%–39%.^11^ Beyond energy intake, these compounds improve insulin sensitivity and reduce chronic low‐grade systemic inflammation and lipotoxicity in metabolic key organs such as the liver and muscle.^12^, ^13^

However, while the benefits of significant FM loss are well established, concerns have been raised regarding the potential unintended loss of SMM and physical function associated with GLP‐1RA‐induced weight reduction. Recent studies have demonstrated that WL achieved through GLP‐1RAs is associated with a variable LBM loss, typically accounting for approximately 20%–40% of the total WL,^14^ hence suggesting a risk of sarcopenia (low muscle mass and impaired muscle function), particularly in populations like elderly individuals and patients with chronic metabolic diseases.^15^ By integrating data from diverse interventions, this narrative review aims to provide a clear understanding of how therapy with GLP‐1RAs can affect muscle mass, underlining research gaps and foreseeing future research areas.

## 2 RESEARCH METHODOLOGY

A comprehensive search was performed in PubMed (coverage through August 2025) focusing on preclinical and clinical studies of incretin‐based therapies and body composition outcomes. Search strings combined controlled vocabulary and keywords, (e.g. ‘GLP‐1 receptor agonist’ or ‘semaglutide’ or ‘liraglutide’ or ‘dulaglutide’ or ‘tirzepatide’ and ‘lean body mass’ or ‘skeletal muscle mass’ or ‘appendicular lean mass’ or ‘muscle strength’ or ‘weight loss’ or ‘muscle preservation’ or ‘body composition’ or ‘diet’ or ‘physical activity’). We screened titles/abstracts to select reports addressing body composition (DXA/BIA/MRI/CT), muscle mass/quality and/or physical function during GLP‐1RA or dual agonist therapy in adults. We included RCTs, controlled trials, prospective/retrospective studies and relevant mechanistic/preclinical work; after removing duplicates, we excluded paediatric studies, case reports and studies without extractable body‐composition or functional outcomes. Full texts were reviewed for population, intervention, duration, methods and outcomes. Given the narrative nature of this review, no formal systematic review protocol or meta‐analysis was conducted. Eligible studies were summarized and synthesized to provide an integrated overview of current evidence. Key references identified during the development of the review, although not emerging from our specific search, were also included when supportive of the topics of interest. Figure 1 and graphical abstract were created with Biorender.com.

## 3 TERMINOLOGY AND MEASURES

In this review we used LBM to denote DXA‐derived lean soft tissue (i.e. fat‐free mass excluding bone mineral content, BMC), which includes skeletal muscle, organs, extracellular water and glycogen. Note that LBM also includes non‐muscle compartments (organs, extracellular water, glycogen) and a small fat‐free fraction of adipose tissue. Consequently, shifts in hydration or glycogen, or method‐specific differences (e.g. DXA vs. BIA), can make LBM appear to change even when true muscle mass does not. SMM, a subset of LBM, refers specifically to the mass of skeletal muscle and can be directly measured through computed tomography (CT) scans or magnetic resonance imaging (MRI). DXA does not directly quantify SMM; instead, it can report appendicular lean mass (ALM) which is the sum of arm and leg lean soft tissue and is often used as a proxy or converted to SMM via validated equations. Many studies label muscle mass as appendicular skeletal muscle mass (ASM). When derived from bioimpedance analysis (BIA), we refer to it as ASM. BIA provides electrical estimates of body compartments and appears frequently in different studies; however, its accuracy is method and hydration‐dependent. Additionally, MRI/CT can also analyse muscle quality and regional muscle size and have been used in clinical trials.

## 4 SKELETAL MUSCLE MICROSTRUCTURE AND QUALITY

Effects of GLP‐1 on muscle microstructure and quality have been studied in preclinical and clinical studies.

Across cell and rodent studies, GLP‐1‐based therapies have been shown to attenuate atrophy programs and support myogenesis. Liraglutide promotes myogenesis via GLP‐1R–dependent cAMP/PKA with downstream PI3K‐AKT and MAPK cascades, also limiting atrophy in freeze‐injury, denervation and dexamethasone models, and improving contractile performance.^16^ Treatment with liraglutide and semaglutide has been shown to decrease atrophy markers via a SIRT1‐dependent mechanism, beyond improved glucose tolerance and insulin resistance, both *in vivo* (high‐fat diet obese mice) and *in vitro* (palmitate‐exposed C2C12 myotubes).^17^ Exendin‐4 (a natural peptide that mimics the effects of GLP‐1) *in vitro,* and dulaglutide *in vivo* model of dexamethasone‐induced atrophy also showed suppression of atrophy genes (e.g. myostatin, atrogin‐1, MuRF‐1) and enhancement of myogenic factors (MyoD, myogenin).^18^ Targets like atrogin‐1/MuRF‐1 regulate ubiquitin‐proteasome‐mediated muscle proteolysis.^19^ In addition, dulaglutide can mitigate disuse atrophy and apoptosis in mice, restoring fibre size and strength and reducing nuclear factor kappa B activation with lower release of proinflammatory cytokines such as TNFα, interleukin (IL)‐1β and IL‐6.^20^ These findings have also been confirmed in aged mice.^21^ In diabetic mice with chronic liver disease, semaglutide preserved grip strength and prevented psoas muscle atrophy; in palmitate‐stressed C2C12 myotubes it suppressed ubiquitin–proteasome–mediated proteolysis and promoted myogenesis via GLP‐1R/cAMP–PKA–AKT signalling, with reduced ROS/inflammation and restoration of mTOR activity.^22^

GLP‐1 exposure also recruits muscle microvasculature in humans (possibly supporting delivery of insulin and nutrients).^23^ Chronic stimulation with liraglutide preserves capillary density and prevents microvascular insulin resistance in high‐fat‐fed rats, increasing AMPK phosphorylation, the expression of VEGF and related receptors and plasma NO content.^24^ GLP‐1 has also been shown to increase skeletal and cardiac muscle microvascular blood volume in humans by 30%–40%, without changing conduit‐artery diameter, consistent with a microvascular rather than large artery effect.^25^ Together, these data support a vascular route by which GLP‐1RA exposure could improve muscle quality.

At mitochondrial level, following a semaglutide‐associated weight loss, skeletal‐muscle mitochondrial efficiency has been reported to improve in obese mice, showing the increase of skeletal‐muscle mitochondrial oxidative phosphorylation (OXPHOS) efficiency, measured as ATP production per O_2_ consumed in permeabilized muscle fibres^26^ (Figure 1). Another preclinical work confirms these findings, suggesting GLP‐1R activation in skeletal muscle in mice promotes an oxidative shift, increasing type 1 fibres and mitochondrial biogenesis.^27^

In conclusion, direct GLP‐1R expression in human skeletal muscle is debated. Hence, many of the preclinical benefits above may arise indirectly, via improved nutrient delivery, enhanced microvasculature, anti‐inflammatory effects and weight loss‐related remodelling. However, translation to humans is not yet established.

## 5 BODY COMPOSITION AND PERFORMANCE OUTCOMES

Across randomized and controlled studies (summarized in Table 1), GLP‐1RAs produce predominant FM loss with a smaller absolute loss of LBM, considering that muscle mass corresponds to about half of LBM.^28^

**TABLE 1:** Summary of representative studies assessing body composition and functional outcomes with GLP‐1–based therapies.

Study (first author, year)	Population	Agent and dose	Duration	Body‐composition method	Main outcomes	Key takeaway
Wilding 2021 (STEP‐1 exploratory)^29^	Adults with overweight/obesity	Semaglutide 2.4 mg QW	68 week	DXA	Larger FM than LBM reduction; relative %LBM increase	Weight loss predominantly from FM; absolute LBM decrease modest
McCrimmon 2020 (SUSTAIN‐8 substudy)^30^	T2D on metformin	Semaglutide 1 mg QW vs. canagliflozin	52 week	DXA	Larger FM than LBM reduction; relative %LBM increase	Pattern FM loss > LBM loss replicated in T2D
Look 2025 (SURMOUNT‐1 subanalysis)^31^	Adults with overweight/obesity	Tirzepatide 5/10/15 mg QW	72 week	DXA	≈75% of weight loss from FM; LBM decline modest	Robust FM loss with relatively smaller LBM loss
Jiao 2025 (meta‐analysis)^32^	Mixed populations	GLP‐1RA (various)	‐	Mixed	Confirms predominant FM loss with modest absolute LBM decline; heterogeneity noted	Synthesis aligns with STEP/SUSTAIN patterns
Osaka 2023 (retrospective)^33^	Older adults with T2D	GLP‐1RA (various) + supervised exercise	9 days	BIA	ASM improved in some patients despite weight loss	Suggests possible ASM preservation in specific settings (non‐randomized)
Ozeki 2022 (pilot)^34^	Adults with obesity and T2D	Semaglutide	26 week	BIA	Weight and FM decreased; LBM/ASM changes small	Small pilot; direction consistent with FM ≫ LBM
Volpe 2022^36^	T2D	Semaglutide	26 week	BIA	Slight reduction in LBM; handgrip maintained	Function preserved despite modest lean mass decline
Ditzenberger 2025^37^	People with HIV and MASLD	Semaglutide	24 week	CT/MRI	Reduced psoas volume with weight loss; chair‐stand/gait speed unchanged	Short‐term weight loss without functional decline
Kosiborod 2023^38^	People with HFpEF and obesity	Semaglutide 2.4 mg	52 week	‐	Higher improvement in the 6‐min walk distance in the semaglutide group vs. placebo	Greater improvement in exercise function

*Note*: Populations, agents/doses, durations, assessment methods (DXA/BIA/MRI/CT) and main findings are listed. Across randomized and controlled settings, fat mass reduction predominates with modest absolute lean mass decreases; functional capacity/strength is generally preserved.

Abbreviations: ASM, Appendicular skeletal muscle mass; FM, Fat mass; LBM, Lean body mass; QW, Once weekly; T2D, Type 2 diabetes.

In the STEP 1 DXA substudy (semaglutide 2.4 mg for 68 weeks vs. placebo), body weight fell ≈15%, with a larger relative reduction in total and visceral FM (−19.3% and −27.4%, respectively) than in LBM (−9.7%), hence increasing the relative LBM/total body mass ratio by ~3%.^29^ In people with T2D on stable‐dose metformin therapy (SUSTAIN‐8 DXA substudy), the proportion of LBM increased by 1.2% after 52 weeks on semaglutide 1 mg.^30^ SURMOUNT‐1 substudy (tirzepatide) reported 33.9% FM and 10.9% LBM reduction at 72 weeks, with 75% of weight lost from FM.^31^ A meta‐analysis of randomized control trials (RCTs) confirms the pattern: GLP‐1‐based therapies substantially reduce FM and only modestly reduce LBM, preserving the %LBM to total body weight similarly to controls.^32^

Short term data also suggest possible attenuation of SMM loss under specific conditions. A small retrospective longitudinal study based on 20 elderly (>70 y/o) hospitalized patients with T2D showed a small but statistically significant increase in ASM in the group treated with GLP‐1RA and insulin (glargine + lixisenatide) in co‐therapy for 9 days (plus physical training 3 times a week) compared to the group treated with insulin only. Body composition was assessed using bioimpedance analysis.^33^ A similar pattern was reported in another retrospective study based on 13 adults (mean age 52 y/o ± 6.9) patients with T2D and obesity: although absolute ASM decreased, semaglutide had no effect on the %ASM after 3 months.^34^

Overall, the rate of weight lost in terms of lean mass varies widely across populations, agents and measurement methods; thus muscle preservation cannot be assumed a priori, especially in older adults or metabolically vulnerable groups. Moreover, equating LBM with SMM can bias interpretation since LBM includes non‐muscle compartments, and a fraction of adipose tissue is FFM. However, when comparing diet, GLP‐1RAs and surgery‐induced WL, absolute LBM losses appear variable but broadly comparable across modalities.^35^

Across the limited clinical literature that measured muscle quality, strength or performance, muscle strength appears preserved and objective functional capacity seems to be stable or improved with GLP‐1RAs. In a 26‐week prospective real‐life study in 40 patients with T2D and obesity, semaglutide reduced FM and visceral adipose tissue with only mild lean/muscle declines and no loss of handgrip strength.^36^ In a single‐arm trial in people with HIV and MASLD, 24 weeks of semaglutide reduced psoas muscle volume (~9%) but chair‐stand times and gait speed did not change significantly, and the prevalence of slow gait dropped from 63% to 46%.^37^ Beyond neutral effects, functional capacity can improve when symptom burden and cardiometabolic inflammation abate: in patients with obesity‐related heart failure with preserved ejection fraction (HFpEF), semaglutide improved by ~20 m the 6‐min walk distance at 52 weeks compared with placebo, along with better symptoms and C‐reactive protein reduction.^38^ In addition, liraglutide has been associated with reduced thigh muscle fat in a randomized trial in a cohort of mostly women with overweight or obesity in the absence of diabetes, improving muscle quality rather than volume.^39^

Notably, the apparent preservation of muscle strength observed with GLP‐1RAs may simply reflect the generic WL beneficial effects (e.g. reduced mechanical load and symptom burden) rather than a direct anabolic effect on skeletal muscle. To date, no randomized or prospective human study has demonstrated a clinically significant increase in measured muscle strength with GLP‐1RAs. Standardized functional batteries (handgrip dynamometry, usual and fast gait speed over 4–6 m, five‐times chair stand and 6‐min walk test where appropriate) should be assessed at baseline and during follow‐up, aligned with body‐composition monitoring (DXA/ALM or CT/MRI where feasible).

## 6 LIFESTYLE INTERVENTIONS: PHYSICAL ACTIVITY AND DIET IN THE CONTEXT OF GLP‐1 THERAPY

As discussed above, different studies suggest a calorie intake reduction during GLP‐1RAs therapy between 16% and 39%^11^; however, muscle mass loss is similar to other obesity therapies such as bariatric surgery and very low‐calorie restricted diets.^40^ Considering the GLP‐1RA‐induced appetite suppression due to the intrinsic effect of the drug and the possible onset of gastroinetstinal side effects, nutritional counselling is generally advised, since a food intake reduction could provoke deficiency of micro‐ and macro‐nutrients, particularly proteins. A retrospective study conducted in a large cohort of people treated with GLP‐1 has shown a 20% incidence of malnutrition after 1 year of treatment.^41^ This aspect gains even more importance when related to the higher risk of malnutrition that people with obesity already have, compared with people with normal body weight of the same age and sex.^42^ To date, specific nutritional aspects have not been properly evaluated and more research is needed to explore changes in the overall dietary intake and eating patterns of people using GLP‐1RAs. However, since protein intake plays a pivotal role in the preservation of LBM, a high protein intake is generally advised, if not contraindicated: 1–1.5 g of protein/kg body weight/day, with an even higher intake for older people or people with comorbidities.^43^

Moreover, correction of nutritional changes and proper protein intake alone may not be sufficient to prevent muscle mass loss, so that physical activity could emerge as relevant. Exercise training still is one of the most effective strategies to mitigate lean mass and strength loss and should be routinely recommended. However, current evidence on patients during GLP‐1Ras‐induced WL is still scarce and not designed around muscle outcomes: after an 8‐week low‐calorie diet, a 1‐year RCT showed that a moderate to vigorous‐intensity exercise program plus liraglutide preserved bone mineral density versus liraglutide alone or exercise alone, but LBM preservation was not a primary endpoint.^44^ Exercise has also been shown to improve weight maintenance: 52 weeks after the cessation of the intervention, the group treated with both liraglutide and supervised exercise showed better control on weight regain and better cardiorespiratory fitness compared to the group treated with liraglutide only.^45^ With semaglutide, a small RCT found that adding 12 weeks of supervised aerobic training in people affected by T2D synergistically improved β‐cell secretory function without superior body‐composition changes versus training alone over that time frame.^46^

## 7 CONCLUSIONS

GLP‐1RA‐induced WL is mostly adipose‐driven, with only a modest reduction in LBM and signs of improved muscle quality and performance. To date, it remains unclear if these improvements should be linked to GLP‐1RAs per se, or to secondary metabolic effects. The variability of methods used for muscle evaluation, the heterogeneity of study and ‘real‐life’ populations, and the different nutritional and physical approaches reported among the different studies, make the interpretation of data even more challenging. Future trials should prespecify muscle‐focused endpoints and incorporate imaging of muscle quality to define optimal mitigation strategies, quantify musculoskeletal effects and identify at‐risk populations.

## AUTHOR CONTRIBUTIONS

Conceptualization, M.S. and G.S.; methodology and literature search, G.De G., G.Di G. and R.B.; data curation and synthesis, G.De.G., M.S. and G.Di.G; writing/original draft preparation, G.De G., G.Di.G. and R.B.; writing—review and editing, M.S.; visualization and figure preparation, M.C.; supervision, G.S. All authors have read and agreed to the published version of the manuscript.

## CONFLICT OF INTEREST STATEMENT

This is an original manuscript and has not been previously published or submitted to another journal. There are no conflicts of interest related to the study design or its results.

## Data Availability

Anonymized data will become available to interested parties for non‐commercial reasons after publication upon reasonable requests made to the corresponding author. Data requestors will need to sign a data access agreement.
